# The freebirth debate: Respecting autonomy while reflecting on our own practices

**DOI:** 10.1111/aogs.70239

**Published:** 2026-04-22

**Authors:** Jóhanna Gunnarsdóttir, Hulda Hjartardóttir

**Affiliations:** ^1^ Faculty of Medicine University of Iceland Reykjavik Iceland; ^2^ Department of Obstetrics and Gynecology Landspitali ‐ the National University Hospital of Iceland Reykjavik Iceland

During the last few years there has been increasing concern among doctors and midwives about a trend called “freebirth.” Advocates of this movement believe that giving birth without the assistance of any health professionals offers women a sense of freedom which will ensure the best outcome for the mother and her baby. This seems to suggest that labor and delivery assistance is in some way constraining women and enforcing procedures that are either unwanted or perceived as unnecessary and harmful. However, it can be questioned whether a woman is truly free if she has been the subject of indoctrination by influencers in a cult‐like fashion and a better term to use could be planned unassisted births. Recent media coverage has unleashed heated debates about the rights of women to choose this option and the right of the child to the best care—even going so far as to suggest this practice needs to be banned.^1^, ^2^ Concern has been raised about the responsibility of those promoting this option and how to deal with false claims of the safety of giving birth unassisted. Although unknown, the risk of adverse neonatal outcome associated with planned unassisted births could be similar as in unexpected births at home or on the way to hospital (unplanned births outside hospital),^3^ or possibly worse, as some women may have declined preceding antenatal care. Obstetricians and midwives can easily anticipate the increased risks associated with a delivery occurring without any professional assistance and may therefore judge the practice of planned unassisted births harshly and use big words to denounce it. The ethics and unclear legal framework for fetal rights was highlighted in a recent AOGS editorial,^2^ and although important to consider, needs to be discussed within each country. However, we also need to understand why some women decide to forgo the safety of a maternity system that is free of charge. Better understanding of the reasons behind this unsafe practice may help us to find solutions that allow us to maintain good maternal and neonatal outcomes of birth.

During the last decades, labor care has changed considerably. In most high‐income settings giving birth at home with assistance from a midwife is not always a part of the model of care that is free of charge.^4^ In the name of safety and practicality, small labor units, both urban and rural, have closed and we have ended up with very large units with thousands of deliveries each year. To further ensure safety, obstetricians, midwives and other colleagues have introduced guidelines and practices to optimize outcomes. There is pressure from the public and from within the professions to ensure that every baby is born in as good a condition as possible. Added to this is the threat of media coverage and legal action if the outcomes are suboptimal. In many countries this emphasis on preventing adverse perinatal outcomes has probably contributed to increased use of interventions such as labor induction and operative deliveries. There have always been critics of this medicalized approach to labor, both from midwives and the women themselves but more rarely from obstetricians, although there is evidence that this is changing.^5^, ^6^ There seems to be a growing call for a different approach to obstetric care—one that reduces systemic pressure to accept labor interventions and instead offers greater choice and respect for individual preferences for birth settings. To follow such trends, the Icelandic Birth Register reports numbers of out of hospital births in the following categories: Births in midwifery‐led units, planned assisted home births, unplanned births and from 2023 planned unassisted births, which may have increased from virtually nonexisting (as far as we know) to 1.4 in 1000 births in 2023 and 2.1 in 1000 births in 2024.^7^ This apparent change raised the awareness in Iceland about this potential challenge and reminded us how quickly trends such as the “freebirth movement” can spread in today's social media‐driven world. Improved registration of births with details about both birth planning and setting of births should be encouraged in all countries.

Healthcare professionals intend to provide the best possible care based on evidence of benefit and harm, taking the parents' views and wishes into account when making shared decisions about birth interventions. Those who work within perinatal care know that parents reporting negative childbirth experiences often feel that their involvement in decision‐making was lacking due to failed communication. Further, qualitative research into motivations to give birth unassisted has found that concern about consent and overuse of interventions is an important reason behind this choice.^8^ Giving birth at a delivery unit can therefore be perceived as unsafe due to fear of being coerced and losing control. This might seem unreasonable to some health care professionals, but such judgments disregard scientific knowledge from the field of psychology, showing that risk perception is not just a matter of facts, but rather a mixture of intuitive reaction and understanding of probabilities.^9^ Indeed, studies have demonstrated that many factors can affect risk perception, such as trust, fairness, control, and choice—all of which are especially relevant to this context.^10^ Obstetricians and midwives agree that the apparent gap in perception of risk should be considered a threat to maternal and perinatal morbidity and mortality. However, we will not bridge this gap by introducing even more fear of harm by manipulating people and labeling the choice of giving birth outside of the hospital setting as irrational and ignorant.^11^ Such an approach is likely to decrease trust in maternity services and drive these individuals further away from accepting care. On the contrary, re‐evaluation of maternity systems is needed, especially the widespread opposition to planned assisted homebirth and midwifery‐led birth units. The available evidence regarding planned low‐risk deliveries suggests similar perinatal outcomes for out‐of‐hospital settings compared with hospital births when attended by well‐educated, skilled midwives working within well‐integrated health systems.^12^, ^13^ Planned deliveries out of the hospital setting are associated with less use of interventions and may reduce operative birth.^14^, ^15^ Most studies' outcomes of births outside the hospital setting exclude unplanned and unassisted births as these are generally regarded as associated with a higher risk of poor outcomes than planned assisted births, a distinction that is important to make when discussing the place of birth.^2^, ^3^

Giving birth is one of the most life‐changing events in every woman's life and satisfaction with the process is therefore of the utmost importance. For many women this means having given birth in an environment where they feel safe. Most women feel this safety in a hospital setting while others feel best at home or in a small unit staffed by midwives. Acknowledging and accepting this choice as a reasonable option in low‐risk pregnancies, instead of judging all births out of the hospital settings as extremely risky, creates a ground for an open dialog that could potentially increase trust in our maternity systems. We need to have an open mind and listen to the needs of women—but how can we improve? Evidence is accumulating regarding parents' understanding of facts pertaining to risk and how much information is necessary regarding the rational aspect of birth choices.^16^ However, more studies are needed on what kind of information parents want to receive and how different presentation of probabilities and framing of facts will make the parents feel, as this may affect their decision and trust. Obstetricians should adapt new strategies which consider the emotional aspect of risk perception and more interdisciplinary research that aims to improve the shared decision‐making process. Such knowledge base could help us to better communicate facts about risk and respectfully consider parents' emotions, which may be necessary to further improve the quality of our consultations. In all these discussions we should remember that the autonomy of women must be respected and thereby their right to decline any investigations, interventions or even birth assistance. Importantly, we must critically investigate our maternity systems to ensure that the information we provide about birth safety and our approach to the birth process will genuinely promote maternal and neonatal well‐being.

## CONFLICT OF INTEREST STATEMENT

The authors declare no conflicts of interest.

## Data Availability

Data sharing not applicable to this article as no datasets were generated or analysed during the current study.
